# Evaluating communication and clinical skills in objective structured clinical examination (OSCE) using a low-cost neck-mounted device, insights from a first-person perspective

**DOI:** 10.1016/j.jds.2025.03.009

**Published:** 2025-03-24

**Authors:** Yu-Ching Liu, I-Ching Liao, Ching-Yi Wu, Chung-Han Ho, Chih-Hao Lu, Kuan-Liang Chen, Chao-An Chen

**Affiliations:** a Division of Periodontics, Department of Dentistry, Chi Mei Medical Center, Liouying, Tainan, Taiwan; b Division of Periodontics, Department of Dentistry, Chi Mei Medical Center, Tainan, Taiwan; c Division of Endodontics, Department of Dentistry, Chi Mei Medical Center, Tainan, Taiwan; d Institute of Oral Biology, National Yang Ming Chiao Tung University, Taipei, Taiwan; e Department of Medical Research, Chi-Mei Medical Center, Tainan, Taiwan; f Institute of Bioinformatics and Systems Biology, National Yang Ming Chiao Tung University, Hsinchu, Taiwan; g Department of Biological Science and Technology, National Yang Ming Chiao Tung University, Hsinchu, Taiwan; h Center for Intelligent Drug Systems and Smart Bio-Devices, National Yang Ming Chiao Tung University, Hsinchu, Taiwan; i Division of Endodontics, Department of Dentistry, Chi Mei Medical Center, Liouying, Tainan, Taiwan

**Keywords:** Cell phone use, Educational measurement, Smartphone, Video recording

## Abstract

**Background/purpose:**

This study used a neck-mounted mobile phone from a first-person perspective to record the performance of dental interns in an objective structured clinical examination (OSCE) to evaluate the effectiveness of video feedback and to assess medical communication and clinical skills.

**Materials and methods:**

Standardized patients wore neck-mounted mobile phones to record the examination process from a first-person perspective (FPP). Pre- and post-test questionnaires measuring anxiety, impacts on communication skills and performance, distraction, technical challenges, feedback received from video, and educational value of the neck-mounted phones were completed by dental interns. The questionnaires were scored on a four-point scale to compare the differences based on gender and between pre- and post-test scores.

**Results:**

This study recruited 30 dental interns to assess the use of neck-mounted phones during an OSCE. The comparison of pre- and post-test questionnaires showed significant improvements in expectations for increased usage and agreements on the value of education and significant differences in reduced distraction and performance concerns. Both male and female post-test groups scored higher with significant improvements. In the post-test questionnaire, female group showed significant differences in perceptions of negative performance impacts and willingness to use this device, while male group showed a significant anxiety reduction.

**Conclusion:**

FPP videos can be used to evaluate oral communication skills in doctor-patient interactions, enhance empathy, and contribute to holistic care.

## Introduction

Objective structured clinical examination (OSCE) is essential for assessing clinical skills, with video recordings serving as valuable educational tools. Traditional third-person perspective cameras often provide limited audio and visual coverage. Wearable devices like Google Glass (Google, Mountain View, CA, USA) and GoPro camera system (GoPro, San Mateo, CA, USA) have addressed these issues, offering high-quality first-person perspective (FPP) vedios.^1^^,^^2^ However, research on wearable devices in OSCE settings is still remains limited.^3^^,^^4^ Studies have shown that using devices like Google Glass to record physicians' performance from a FPP can enhance communication skills and empathy.^4^ By allowing observation of facial expressions and body language from the patient's perspective, these devices have proven valuable for clinical skills education.^3^

FPP videos play a crucial role in medical education, particularly in enhancing learning outcomes through immersive and authentic learning experiences. FPP videos offer a unique perspective that allows students to observe clinical scenarios from a patient's viewpoint. This perspective helps improve decision-making, non-technical skills, and crisis management abilities by providing a clearer understanding of real-time interactions. FPP videos also facilitate detailed feedback and self-reflection, making them valuable for improving communication skills, empathy, and overall clinical competence.^5^^,^^6^ Previous research has shown that FPP videos can promote active engagement, help students reinforce their memory of clinical procedures, and support the development of complex skills. Their application in simulation training for clinical skills, such as cardiopulmonary resuscitation (CPR), further highlights their ability to enhance both technical and non-technical performance.^7^

In the past, wearable recording devices primarily utilized Google Glass for clinical education in medical communication and clinical skills.^2^^,^^8^ However, its high cost, limited battery life, and accessibility challenges have restricted its widespread application in resource-limited environments. In contrast, smartphones have become an integral tool in medical education, particularly in OSCE, due to their versatility and accessibility. Using smartphones for video recording, real-time assessment, and self-evaluation has gained popularity as an alternative to traditional methods.^9^, ^10^, ^11^ Smartphones help students record and review performance, enhancing clinical skills, communication, and self-directed learning. They boost self-efficacy, learning satisfaction, and engagement through repeated practice. Their portability and cost-effectiveness suit resource-limited settings, aligning with learner-centered medical and dental education, making them valuable for OSCE assessments.^9^ Furthermore, a neck-mounted smartphone system was developed to address the inconvenience of holding smartphones during teaching. This system retains the FPP advantages of Google Glass while offering better cost-effectiveness and longer battery life. This hands-free solution allows instructors to teach flexibly without frequent charging, making it a practical and scalable tool for enhancing medical education and clinical training.

Regarding the potential advantage of a hand-free device in OSCE teaching and assessment, this innovative educational study aimed to determine acceptance of FPP videos recorded by the neck-mounted smartphone system during the exam by dental interns, who must pass the OSCE before completing their internship at our hospital. The effectiveness of the device on communication and clinical skills was also studied.

## Materials and methods

**Figure 1:**
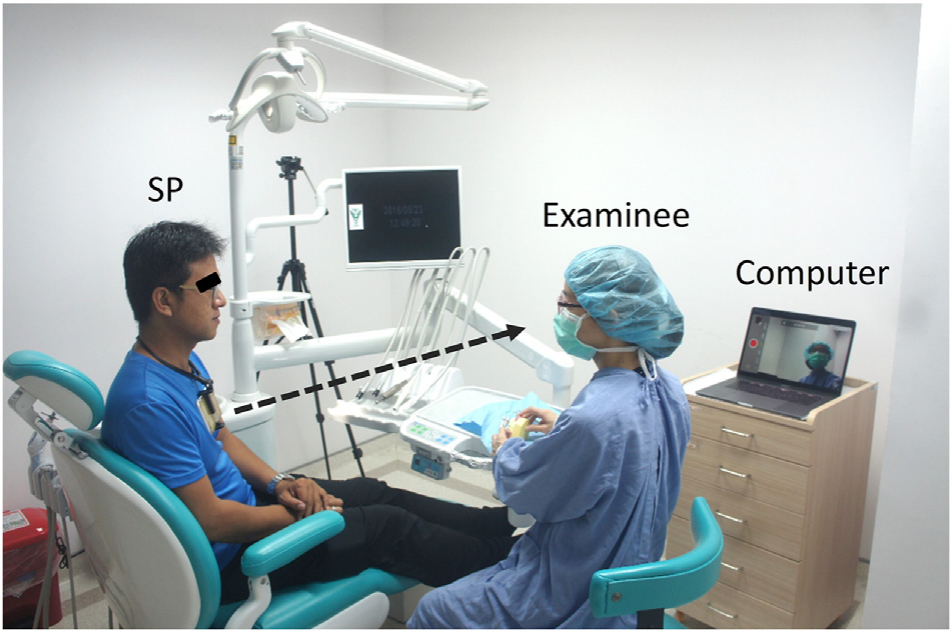
A standardized patient (SP, left) could be synchronized with a computer screen using a neck-mounted mobile phone to record the facial expressions and responses of examinees, eliminating the need for staff to manipulate video cameras.

This study received approval from the Research Ethics Committee of Chi-Mei Hospital (reference number: 11111-L04). OSCE assessment was conducted on dental interns. Standardized patients (SPs) wore neck-mounted mobile phones installed with ApowerMirror Screen Recorder software (Wangxu Technology CO., Limited, Hong Kong, China), which synchronized the phone screen to a nearby computer monitor. SP could use the mirror image to adjust the angle of the mobile phone to optimize the video quality of the FPP recordings. Then SP captured the examinee's facial expressions, body movements, and speaking volume with the neck-mounted mobile phones (Fig. 1).

Before the examination, the students watched instructional videos and were informed that the standardized patients would be wearing neck-mounted mobile devices to record the examination process. They were also asked to complete a pre-test questionnaire. After the examination, the students watched recorded videos from the mobile phones and received feedback from the instructors. They then completed a questionnaire that was the same as the pre-test for the post-test questionnaire. The questionnaire was developed and implemented according to modified criteria derived from previous research.^4^ The student questionnaires consisted of nine items: (1) I do not feel anxious about SP wearing a neck-mounted mobile phone during the procedure. (2) During the procedure, using a neck-mounted mobile phone by SP effectively improves my communication skills. (3) The neck-mounted mobile phone distracts me. (4) Knowing the video recordings from the neck-mounted mobile phones has a negative impact on my performance. (5) Using neck-mounted mobile phones is technically challenging. (6) The feedback I receive from neck-mounted mobile phones is helpful. (7) I feel that the recorded images from the neck-mounted mobile phones provide me with opportunities to receive feedback that I had not received before. (8) I look forward to having more opportunities to use neck-mounted mobile phones. (9) I understand the value of neck-mounted mobile phones in medical education.

The questionnaires were scored on a four-point scale: strongly disagree (one point), disagree (two points), agree (three points), and strongly agree (four points). The agreement rate (%) and the weighted mean of each item were calculated for the questionnaire. The agreement rate was calculated as (strongly agree + agree)/number of students x 100 %. The difference between pre- and post-test questionnaires was estimated using the paired sample t-test or Wilcoxon signed rank test depending on the normality tests. Additionally, the difference between male and female groups was compared using the Mann–Whitney U test.^4^

## Results

Thirty dental interns participated in the OSCE, including 14 males (46.7 %) and 16 females (53.3 %). According to the pre-test questionnaire, 63.3 % of the interns did not feel anxious about SPs wearing neck-mounted phones, while only 30 % reported technical difficulties in utilizing the neck-mounted phones. Furthermore, 40 % believed the devices could improve communication skills, but 66.7 % found them distracting, and 50 % thought they negatively impacted performance. Between 73.3 % and 83.3 % of the interns appreciated the video feedback from the recordings, recognized its educational significance, and considered it an opportunity for additional feedback. Approximately half expressed interest in using neck-mounted phones more frequently (Table 1).

**Table 1:** Survey and comparison Mean score of pre-test and post-test (n = 30).

Statement		Agreement (%)	Mean ± SD	*P*-value^a^
1. I do not feel anxious about SP wearing a neck-mounted phone during the procedure.	Pre-test	63.3	2.57 ± 0.63	0.071
	Post-test	70.0	2.87 ± 0.68	
2. During the procedure, using a neck-mounted mobile phone by SP effectively improves my communication skills.	Pre- test	40.0	2.43 ± 0.57	0.839
	Post-test	43.3	2.47 ± 0.78	
3. The neck-mounted phone distracts me.	Pre-test	66.7	2.77 ± 0.63	0.016∗∗
	Post-test	43.3	2.43 ± 0.63	
4. Knowing the video recordings from the neck-mounted mobile phones has a negative impact on my performance.	Pre-test	50.0	2.47 ± 0.57	<0.001∗∗
	Post-test	16.7	2.10 ± 0.61	
5. Using neck-mounted phones is technically challenging.	Pre-test	30.0	2.27 ± 0.52	0.070
	Post-test	13.3	2.03 ± 0.61	
6. The feedback I receive from neck-mounted phones is helpful.	Pre-test	76.7	2.80 ± 0.48	0.070
	Post-test	90.0	3.03 ± 0.49	
7. I feel that the recorded images from the neck-mounted mobile phones provide me with opportunities to receive feedback that I had not received before.	Pre-test	83.3	2.90 ± 0.48	0.070
	Post-test	93.3	3.13 ± 0.51	
8. I look forward to having more opportunities to use neck-mounted mobile phones.	Pre-test	50.0	2.50 ± 0.51	0.003∗∗
	Post-test	80.0	2.93 ± 0.58	
9. I understand the value of neck-mounted phones in medical education.	Pre-test	73.3	2.80 ± 0.55	0.012∗∗
	Post-test	93.3	3.20 ± 0.55	

**SP**, Standardized Patient. Agreement is represented of ‘’strongly agree’’ or ‘’agree’’ on a four-point scale including Strongly agree (four points), Agree (three points), Disagree (two points), and Strongly disagree (one point)**.**

∗∗*P* < 0.05 means significant difference.

a Paired sample t test.

Post-test results indicated improvements: 70.0 % of the interns reported no anxiety about the neck-mounted phones, and only 13.3 % encountered technical difficulties. Additionally, 43.3 % believed the devices enhanced communication skills and found them distracting, and 16.7 % felt their performance was negatively impacted. Between 80.0 % and 93.3 % valued the feedback provided, recognized the educational value and 80.0 % expressed a desire for more opportunities to use the device (Table 1).

Comparing pre-test and post-test results revealed significant improvements in several areas, including reduced anxiety, improved communication skills, greater appreciation for video feedback, and increased willingness to use neck-mounted phones. Significant differences (*P* < 0.05) were noted in the perception of the device's value in medical education and the desire for continued use. Conversely, perceptions of distraction, negative performance impact, and technical difficulties decreased significantly (*P* < 0.05) (Table 1).

Mean scores improved significantly from 23.50 ± 2.81 pre-test to 26.07 ± 2.97 post-test (*P* < 0.05). Both male and female groups scored higher on the post-tests, and both groups showed significant differences (*P* < 0.05) (Table 2).

**Table 2:** Difference of total mean standard score of pre-test and post-test.

	Pre-test	Post-test	*P*-value^a^
**Total score**	23.50 ± 2.81	26.07 ± 2.97	<0.001∗∗
**Sex**			
Female (n = 16)	23.19 ± 2.88	25.50 ± 2.99	0.011∗∗
Male (n = 14)	23.86 ± 2.80	26.71 ± 2.92	0.023∗∗

**∗∗***P* < 0.05 means significant difference.

a Paired sample t test.

Gender analysis showed that pre-test, male interns scored higher on five items: performance improvement, technical difficulties, new feedback from recordings, expectations of using the device, and educational value. However, these differences were not statistically significant. In the post-test, males scored higher on items related to comfort with SPs using the devices, helpfulness of video feedback, new feedback opportunities, expectations for device use, and educational value, though these differences also lacked statistical significance (Table 3).

**Table 3:** Comparison mean scores of pre-test and post-test between female and male for each questionnaire.

Statement	Pre-test			Post-test		
	Female Mean ± SD	Male Mean ± SD	*P*-value∗	Female Mean ± SD	Male Mean ± SD	*P*-value∗
1. I do not feel anxious about SP wearing a neck-mounted phone during the procedure.	2.69 ± 0.48	2.43 ± 0.76	0.391	2.69 ± 0.70	3.07 ± 0.62	0.118
2. During the procedure, using a neck-mounted phone by SP effectively improved my communication skills.	2.38 ± 0.50	2.50 ± 0.65	0.680	2.50 ± 0.82	2.43 ± 0.76	0.874
3. The neck-mounted phone distracts me.	2.88 ± 0.62	2.64 ± 0.63	0.311	2.56 ± 0.51	2.29 ± 0.73	0.166
4. Knowing the video recording from neck-mounted phone has a negative impact on my performance.	2.56 ± 0.63	2.36 ± 0.50	0.238	2.13 ± 0.50	2.07 ± 0.73	0.592
5. Using neck-mounted phones is technically challenging.	2.25 ± 0.58	2.29 ± 0.47	0.940	2.06 ± 0.68	2.00 ± 0.55	0.979
6. The feedback I receive from neck-mounted phones is helpful.	2.81 ± 0.54	2.79 ± 0.43	0.957	3.00 ± 0.52	3.07 ± 0.47	0.715
7. I feel that the recorded images from the neck-mounted mobile phones provide me with opportunities to receive feedback that I had not received before.	2.81 ± 0.54	3.00 ± 0.39	0.285	3.06 ± 0.57	3.21 ± 0.43	0.468
8. I look forward to having more opportunities to use neck-mounted mobile phones.	2.44 ± 0.51	2.57 ± 0.51	0.487	2.88 ± 0.62	3.00 ± 0.55	0.566
9. I understand the value of neck-mounted phones in medical education.	2.75 ± 0.58	2.86 ± 0.53	0.598	3.13 ± 0.50	3.29 ± 0.61	0.407

∗ *P*-value of Mann–Whitney U test.

Post-test data revealed that three items showed score reductions across genders: distraction, negative performance impact, and technical challenges in using the neck-mounted smartphone. Among females, significant improvements were observed in the perceived performance impact (2.56 ± 0.63 vs. 2.13 ± 0.50, *P* < 0.05) and willingness to use the device (2.44 ± 0.51 vs. 2.88 ± 0.62, *P* < 0.05). Among males, a significant reduction in anxiety about using the device was noted post-test (*P* < 0.05) (Table 4).

**Table 4:** Compare pre-test and post-test scores in female group and male group.

Statement	Female (n = 16)			Male (n = 14)		
	Pre-test Mean ± SD	Post-test Mean ± SD	*P*-value^a^	Pre-test Mean ± SD	Post-test Mean ± SD	*P*-value^a^
1. I do not feel anxious about SP wearing a neck- mounted phone during the procedure.	2.69 ± 0.48	2.69 ± 0.70	>0.99	2.43 ± 0.76	3.07 ± 0.62	0.031∗∗
2. During the procedure, using a neck-mounted phone by SP effectively improved my communication skills.	2.38 ± 0.50	2.50 ± 0.82	0.781	2.50 ± 0.65	2.43 ± 0.76	>0.99
3. The neck-mounted phone distracts me.	2.88 ± 0.62	2.56 ± 0.51	0.188	2.64 ± 0.63	2.29 ± 0.73	0.188
4. Knowing the video recording from neck-mounted phone has a negative impact on my performance.	2.56 ± 0.63	2.13 ± 0.50	0.016∗∗	2.36 ± 0.50	2.07 ± 0.73	0.219
5. Using neck-mounted phones is technically challenging.	2.25 ± 0.58	2.06 ± 0.68	0.453	2.29 ± 0.47	2.00 ± 0.55	0.313
6. The feedback I receive from neck-mounted phones is helpful.	2.81 ± 0.54	3.00 ± 0.52	0.531	2.79 ± 0.43	3.07 ± 0.47	0.219
7. I feel that the recorded images from the neck-mounted mobile phones provide me with opportunities to receive feedback that I had not received before.	2.81 ± 0.54	3.06 ± 0.57	0.359	3.00 ± 0.39	3.21 ± 0.43	0.375
8. I look forward to having more opportunities to use neck-mounted mobile phones.	2.44 ± 0.51	2.88 ± 0.62	0.016∗∗	2.57 ± 0.51	3.00 ± 0.55	0.172
9. I understand the value of neck-mounted phones in medical education.	2.75 ± 0.58	3.13 ± 0.50	0.109	2.86 ± 0.53	3.29 ± 0.61	0.172

**∗∗***P* < 0.05 means significant difference.

a **Wilcoxon signed rank**.

## Discussion

OSCE provides unique systematic feedback to students,^12^ and feedback is essential to the dental OSCE learning process. It aims to provide learners with information to reflect on their performance and to plan improvements in their future clinical performance.^13^^,^^14^ Recording is a crucial component of OSCE assessments, as it allows students to observe their own performance and enables instructors to provide timely feedback. High-quality feedback has been identified as one of the main characteristics of any effective learning experience.^15^ National OSCE test centers have used traditionally third-person perspective recording systems to observe student performance. However, these systems sometimes result in poor audio quality and are unable to clearly capture students' facial expressions and body language. Moreover, teachers face challenges in providing detailed, timely, and meaningful feedback to each student.^16^ This limitation can hinder a comprehensive evaluation of a student's communication skills and non-verbal cues such as body language, which are essential aspects of clinical competence.

Both written and audio feedback were reported as meaningful learning sources in OSCE performance, either from students' (93 % written and 89 % audio) or examiners’ (96 % written and 92 % audio) perspectives of views.^17^ Personalized written feedback must be completed in a very short 1–2 min after each station test. In contrast, examiners have more time to gather generalized audio feedback at each station. Our study provided students with video recordings and written feedback after the test, leading to a more complete feedback experience than previous methods.

Recording students' performance with a wearable smartphone enables detailed behavior and communication skills reviews, enhancing learning through accurate reflections for self-assessment, feedback, and peer review. However, some students felt that wearable phones distracted them during the test, and in turn, affected their communication with SPs. To address this, we suggested helping students familiarize themselves with the device and get accustomed to it before the test. For instance, allowing students to watch pre-recorded videos and using the device beforehand could help.^18^^,^^19^

Gender differences in OSCE performance are notable. Female students often excel in communication but report lower confidence levels compared to males.^20^, ^21^, ^22^ In our study, female participants initially felt the neck-mounted phones negatively impacted their performance, reflecting confidence issues. Setting a camera for recording during OSCE assessment might negatively impact performance, and some students felt more anxious when being filmed.^23^ Male students reported higher pre-test anxiety but adapted more quickly post-test, likely due to greater familiarity with technology. As a result, male students perceived the device as having less impact on their focus and communication skills during the test. Differences in technology acceptance between males and females has been observed for decades, although the gap in between has been minimally reduced within the past decades.^24^ Female students are more inclined to adopt educational smartphone applications due to perceived benefits and compatibility with their learning style but often struggle with technical challenges and lower confidence.^25^ Males, in contrast, adapt more quickly and experience less anxiety after initial exposure. While females may initially find new technology overwhelming, targeted interventions—such as pre-training and confidence-building exercises—can improve their adaptability.^26^ Regarding different dimensions of attitudes toward tecnnology, differences in affect (feeings about technology, including anxiety) and self-efficacy (“peoples' belief in their own abilities to undertake a technology-related task successfully”), but not belief (“perceptions and cognitions about technology use and its societal function”), between genders has been significantly reduced during the past decades.^24^ Thus, to address gender differences, future research should explore gender-specific strategies focusing on technology belief, including introductory technical training, real-time support, and peer collaboration. Establishing a supportive learning environment can help mitigate self-confidence issues and ensure equal engagement with educational technologies like smartphones.^25^

Generation Z (Gen-Z) dental interns, typically born between 1995 and 2009, exhibit distinct characteristics that shape their learning preferences and behaviors.^27^ As digital natives, they have grown up with smartphones, tablets, and constant internet connectivity, making technology an essential part of their daily lives. This e-learning generation is accustomed to instant access to information, multitasking, and social media interactions. These characteristics influence their preferred learning methods and engagement in education.^28^ Given their familiarity with digital tools, smartphones naturally serve as an effective medium for teaching, especially in clinical skill assessments. Today's Gen-Z students prefer instant answers through search engines and videos over traditional reading assignments and lectures. As educators, we must adapt our teaching methods to optimize the learning experience for these entrusted students. Our teaching methods must be efficient and customized, while traditional approaches must blend with innovative techniques to meet the needs of Gen-Z learners.^29^

Traditionally, OSCE assessments relied on specialized test centers with professional recording equipment. Aside from the high cost of equipment, unclear video footage and indistinct audio posed challenges for OSCE assessments.^30^^,^^31^ The COVID-19 pandemic has emphasized the need for low-contact learning and assessment in dental education.^32^^,^^33^ As a result, online OSCE models have been developed, and real-time video recording using smartphones can serve as an effective tool for future online OSCE.^34^, ^35^, ^36^, ^37^ Wearable smartphone devices have emerged as a user-friendly, cost-effective solution with excellent video recording capabilities, enabling flexible, remote assessments that reduce physical interaction while maintaining high quality evaluations. Clinical procedures can be recorded and evaluated in real-time, allowing examiners to observe and score performances remotely, removing the need for centralized testing facilities. This approach not only ensures safety but also offers flexibility in conducting assessments in various settings. In this study, we used the ApowerMirror Screen Recorder to synchronize the computer and smartphone screens, allowing standardized patients to adjust the camera and track the examinee's facial expressions and procedures directly from the computer screen, thus eliminating the need for additional personnel to record. Furthermore, examiners can view and score the performance via the synchronized smartphone footage.

Although Gen-Z dental interns are familiar with technology and have adapted quickly, utilizing video-based feedback to develop clinical skills. However, there were some limitations in this study. Several smartphone models are heavy leading to their slipping or shifting during wear. Proper securing is necessary to avoid disrupting the camera angle. Tighten the straps or clamps and ensure proper alignment to evenly distribute the phone's weight or add anti-slip materials such as thin rubber or silicone pads along with a protective case to prevent sliding. Additionally, the study involved only 30 participants, the sample size was small. The participants were also restricted to interns. Future research should include more participants and categorize them into interns, first year of post-graduate (PGY1), and second year of post-graduate (PGY2) residents to provide a more comprehensive analysis.

The single-group pretest-posttest design of the study allowed us to minimize variability from gender and prior experiences. However, the absence of a control group made a direct comparison between neck-mounted devices and traditional methods impossible. Regarding the fact that all dental students must pass OSCE before completing their internship, the exam's fairness makes the design of a control group unethical and impractical. Thus, further study should be conducted in a scenario that will not interfere with an examination's outcome to include a control group. On the other hand, gender differences in device acceptance may be associated with multiple factors, including psychological, sociocultural, or technical background factors. In the future in-depth study, questionnaires that approach these dimensions should be considered.

In conclusion, through FPP video feedback, students can observe their facial expressions, body language, and vocal tone. This method aids in evaluating verbal communication skills and can enhance empathy, ultimately contributing to better holistic patient care.

## Declaration of competing interest

The authors have no conflict of interest relevant to this article.
